# Hyperbaric therapy provides no benefit for skeletal muscle and respiratory function and accelerates cardiac injury in mdx mice

**DOI:** 10.1038/s41598-019-48744-7

**Published:** 2019-08-23

**Authors:** Kaleb D. Fischer, Jackie A. Heitzman, DeWayne Townsend

**Affiliations:** Department of Integrative Biology and Physiology, Medical School, University of Minnesota, Minneapolis, MN USA

**Keywords:** Cardiomyopathies, Neuromuscular disease

## Abstract

Duchenne muscular dystrophy (DMD) is a uniformly fatal condition of striated muscle wasting resulting in premature death from respiratory and/or cardiac failure. Symptomatic therapy has prolonged survival by limiting deaths resulting from respiratory insufficiency, but there is currently no effective therapy for most patients with DMD. This grim prognosis has led patients and their families to seek unproven therapeutic approaches. One such approach is the use of hyperbaric therapies, which 14% of DMD patients self-report using. The primary goal of this study was to determine if intermittent hyperbaric exposure altered the muscle function of the mdx mouse, a genetic model of DMD. To do this, mdx mice were exposed to three daily 90-minute 1.3 atmosphere hyperbaric exposures for 4 weeks. Skeletal muscle, respiratory, and cardiac function were assessed in treated and untreated wild type and dystrophic mice. The results of these studies find that hyperbaric and hyperoxic approaches resulted in increased cardiac fibrosis in dystrophic mice and no beneficial effects on the functional parameters measured. These data suggest that these oxygen-based therapies are unlikely to provide therapeutic benefit to DMD patients.

## Introduction

Duchenne muscular dystrophy (DMD) is a devastating disease characterized by muscle wasting, respiratory insufficiency, and cardiomyopathy^1–3^. This disease is uniformly fatal and there are currently no therapies that are effective for all DMD patients. This has resulted in the pursuit of a wide variety of alternative therapies by patients and their families. Registry data of DMD patients reveals that 14% of patients are using hyperbaric therapies^4^, despite the lack of evidence supporting a therapeutic benefit. Given that patients and their families are already utilizing this approach, we undertook this study to assess the efficacy of hyperbaric exposure in a mouse model of DMD.

Hyperbaric oxygen therapy consists of placing patients into environments with elevations in barometric pressure, sometimes with additional supplemental oxygen. This increases the concentrations of oxygen available to the body. Hyperbaric therapy has FDA approval for a wide variety of disease conditions, most notably decompression syndrome^5^, carbon monoxide poisoning^6^, and poorly vascularized inflammatory conditions (e.g. crush injuries), especially when complicated by anaerobic bacterial infection^7–9^. Exposure to a hyperbaric environment increases the level of oxygen dissolved within the blood, but notably it does not have a dramatic effect on overall oxygen carrying capacity.

To our knowledge, there is no evidence supporting or refuting the efficacy of hyperbaric therapy in DMD. Despite this, hyperbaric therapy is being pursued by many DMD patients. In addition to questions regarding the efficacy of hyperbaric therapies, there remain significant questions about the overall safety of this approach. It is hypothesized that any beneficial effects of hyperbaric exposure will be the result of increased oxygen delivery. To control for this possibility, we have included an experimental group in which only the levels of ambient oxygen are changed. The study reported here assessed the effect of 4 weeks of hyperbaric or hyperoxic therapy on the skeletal muscle, respiratory muscle, and cardiac function in the mdx mouse.

## Results

### Hyperbaric and hyperoxia therapeutic approaches

**Figure 1:**
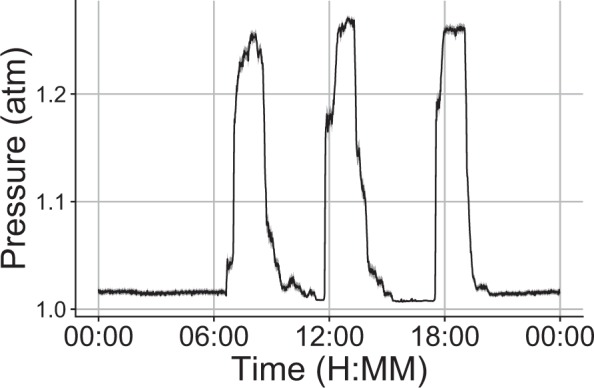
Average daily pressure readings within the hyperbaric chamber. Mean pressure recordings from within the hyperbaric chamber. Data recorded every minute, mean in black, standard error of the mean in grey is derived from data collected from 57 days of the hyperbaric protocol.

C57BL/10 and mdx mice were subjected to 4 weeks of hyperbaric, hyperoxia, or standard room conditions. All mice were housed in the same room to control for environmental factors that might contribute to the progression of the disease. The goal of this study was to test the hypothesis that increases in oxygen delivery, either by hyperbaric approaches or increasing the concentration of environmental oxygen, would improve the dystrophic phenotype. To achieve this, mice were subjected to three 90-minute periods of hyperoxia/hyperbaric conditions each day (Fig. 1). These periods occurred at set times during the day throughout the protocol. This protocol represents a high degree of hyperbaric exposure and is similar to protocols recommended to DMD patients. Hyperbaric chamber pressure and hyperoxic chamber oxygen levels were computer controlled with monitoring throughout the protocol to ensure that the degree of exposure was properly matched.

### Effect of oxygen therapy on dystrophic skeletal muscle function

**Figure 2:**
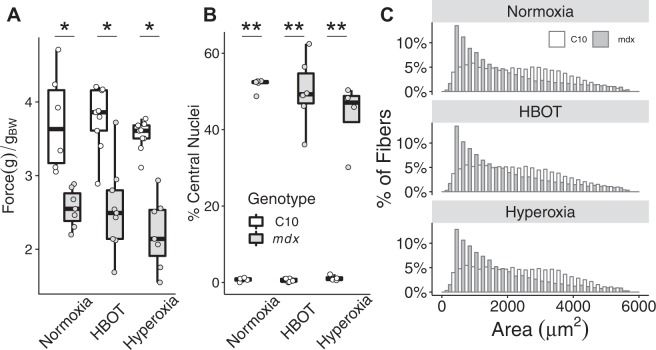
Grip strength and histological assessments of C10 and mdx mice following 4 weeks of oxygen based treatments. (**A**) Mean whole body grip strength, (**B**) percentage of quadricep fibers with central nucleation, and (**C**) the distributions of quadricep cross-sectional area from wild type (C10) and dystrophic (mdx) mice after normobaric, hyperbaric, or hyperoxic exposure for 4 weeks. *P < 0.001, **P < 0.0001.

**Figure 3:**
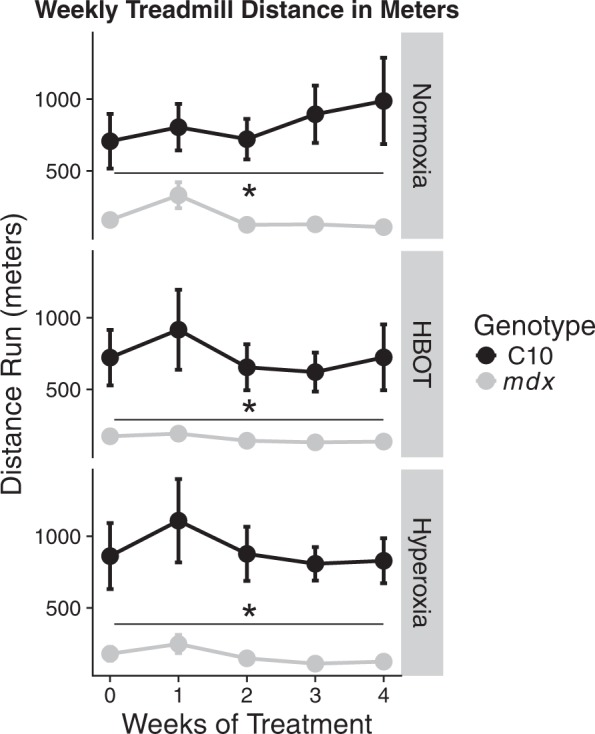
Distance run by C10 and mdx mice on down-hill treadmill. Distance run on downhill treadmill protocol. *Represents three-way ANOVA indicating a significant difference (P < 0.001) between C10 and mdx mice, data represents the mean ± standard error of the mean, treatment groups of 5–7 mice.

Whole body grip strength is an assay that provides a measure of global muscle strength in the mouse. This assay demonstrated a significant reduction in strength in mdx mice (Fig. 2A). The weakness observed in dystrophic mdx mouse muscle was not significantly altered by any of the oxygen based therapeutic approaches (Fig. 2A). Oxygen therapies also had no significant effect on quadricep fibrosis, central nucleation, or fiber cross-sectional area distribution in mdx or C10 mice (Fig. 2B,C), although highly significant genotype effects are present. Downhill running is another assay in which mdx mice perform significantly worse than wild type mice. Similar to the results from the hand grip assay, the performance of mdx mice on the treadmill was not significantly improved by hyperbaric or hyperoxic therapeutic modalities (Fig. 3).

### Effects of oxygen therapy on respiratory function

**Figure 4:**
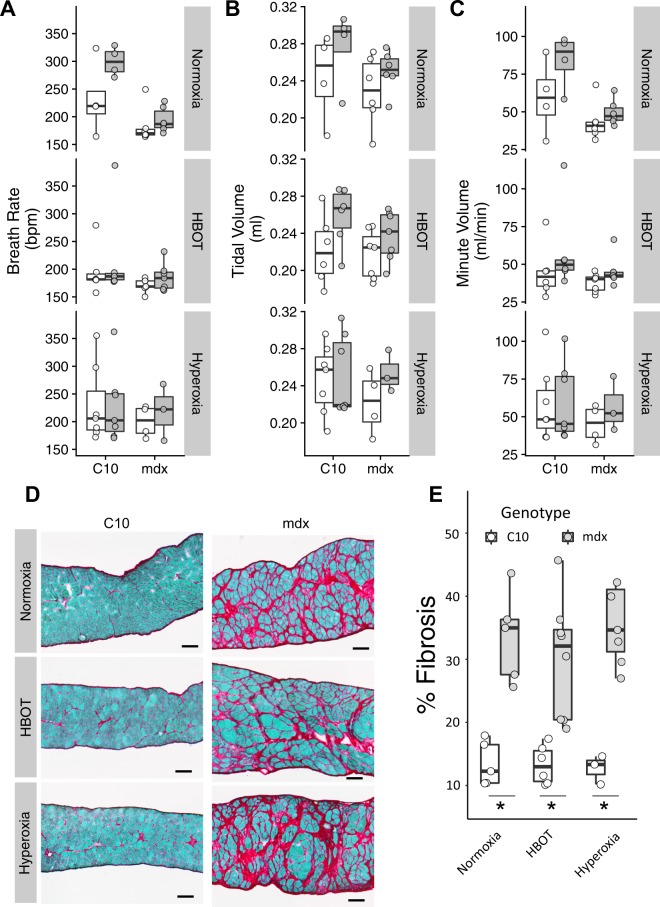
Whole body plethysmography data from C10 and mdx mice following four weeks of oxygen based treatments. Measures of global respiratory function before (white) and after (grey) four weeks of hyperbaric or hyperoxic treatment. Breath rate (**A**), tidal volume (**B**), and minute volume (**C**) are shown. There is no significant treatment effect on the histopathological appearance of the diaphragm muscle (**D**,**E**). Group sizes range from 3–7 mice, no significant treatment effects are observed with a three-way ANOVA. Bar represents 100 µm.

Respiratory failure is a leading cause of death in DMD and any therapy that improves respiratory function would be of great value. The use of whole body plethysmography provides a means to assess the respiratory function of conscious mice. Data were analyzed using a three-way ANOVA with genotype, time, and treatment as factoring groups. This analysis revealed a significant genotype effect for both minute volume and breathing frequency (P < 0.01). This effect was independent of any treatment protocol and is present in mice prior to beginning the experimental treatment, indicating that dystrophic mice have lower resting respiratory rates relative to wild type mice. Histopathological assessment of diaphragm fibrosis also found no effect of treatment on the degree of pathology present in the dystrophic diaphragm (Fig. 4).

### Effects of oxygen therapy on cardiac function

**Figure 5:**
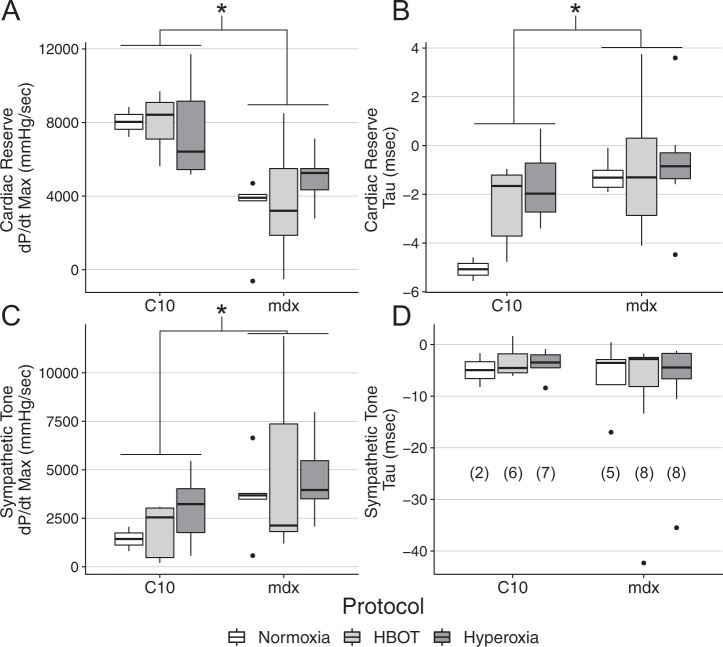
Measures of cardiac reserve and basal sympathetic tone in wild type and dystrophic hearts following 4 weeks of oxygen based therapy. The cardiac reserve (**A**,**B**) is determined during the infusion of 20 µg/kg/min of dobutamine and is expressed as the change above baseline levels. Sympathetic tone (**C**,**D**) is determined from the change from baseline levels to that during 250 µg/kg/min infusion of esmolol. The systolic (dP/dt_Max_) and diastolic (Tau) measures of cardiac reserve display a significant genotype effect, but there is no effect of the treatment protocol on cardiac reserve. The basal systolic function of the dystrophic heart is supported by a greater level of sympathetic tone relative to wild type hearts. The effect of removing sympathetic tone on diastolic function is not different between genotypes. The treatment protocol does not have an effect on basal sympathetic tone. Group sizes are listed in panel D. *P < 0.05.

Cardiac dysfunction is very common in patients with DMD and mdx mice have reductions in cardiac reserve^10–13^. To assess the effects of oxygen therapies on dystrophic cardiac function, cardiac catheterization was employed following 4 weeks of either hyperbaric therapy, hyperoxic therapy, or no therapy. The cardiac catheterization protocol consisted of three distinct treatments; baseline conditions, maximal contractile function caused by β-adrenergic receptor stimulation by dobutamine infusion, and measures of contractility in the presence of β-adrenergic receptor blockade with esmolol infusion. These data are provided in Tables 1–6. There are several parameters in which genotype effects are present, these include heart rate, systolic pressure, and minimum pressure derivative. As demonstrated in Tables 1–6, there are also parameters that have main effect differences based on the treatment protocol. Wild type hearts demonstrate significantly greater response to β-adrenergic stimulation by dobutamine in both systolic (Fig. 5A) and diastolic (Fig. 5B) parameters of cardiac function. None of these parameters were significantly altered by the oxygen therapy protocol. Similarly, the lack of a difference in the magnitude of response to the blockade of the β-adrenergic receptor by the antagonist esmolol, demonstrates that mdx mice have greater dependence on endogenous β-adrenergic receptor activation tone for maintenance of baseline systolic function (Fig. 5C). No differences were observed in measures of diastolic function. These studies demonstrate that the oxygen therapies implemented in these studies have no significant effects on baseline cardiac function, cardiac reserve, or the level of adrenergic receptor activation to support cardiac function.

**Table 1:** Normoxic C10 Hemodyanmic Data.

	Baseline	Dobutamine	Esmolol	Significance
Heart Rate (*bpm*)	380 ± 0.52 (2)	536 ± 2.41 (2)	384 ± 2.63 (2)	c
End-Systolic Pressure (mmHg)	79.3 ± 5.21 (2)	100 ± 0.11 (2)	62 ± 14.4 (2)	c,e
End-Diastolic Pressure (mmHg)	12.4 ± 4.7 (2)	13.2 ± 7.15 (2)	11.5 ± 2.06 (2)	
Maximum dP/dt (mmHg/s)	4804 ± 529 (2)	12839 ± 1339 (2)	3373 ± 1157 (2)	a,e
Minimum dP/dt (mmHg/s)	4434 ± 1120 (2)	10208 ± 628 (2)	3130 ± 1572 (2)	c,d,e
Tau (msec)	11.5 ± 2.21 (2)	6.46 ± 1.73 (2)	16.5 ± 5.48 (2)	
End-Diastolic Volume (μl)	59.6 ± 5.53 (2)	62 ± 14.9 (2)	66.2 ± 6.21 (2)	
End-Systolic Volume (μl)	43.8 ± 3.63 (2)	29.9 ± 13.3 (2)	53.8 ± 9.23 (2)	
Cardiac Output (μl/min)	8129 ± 635 (2)	20915 ± 143 (2)	7341 ± 1644 (2)	
PRSW (mmHg⋅μl/μl)	72.4 ± 14.8 (2)	89.6 ± 36.2 (2)	24.1 ± 20.4 (2)	

Significant testing by three-way ANOVA with genotype, oxygen therapy protocol, and hemodynamic protocol treatment as factors with Tukey post-hoc test. a; signifies difference between baseline and dobutamine with normoxic protocol in C10 mice; b: signifies difference between baseline and esmolol with normoxic treatment in C10 mice; c: signifies an overall genotype effect; d: represents an overall difference between normoxia and HBOT treatment; e: represents an overall difference between normoxia and hyperoxic treatment.

**Table 2:** Normoxic mdx Hemodyanmic Data.

	Baseline	Dobutamine	Esmolol	Significance
Heart Rate (*bpm*)	490 ± 22.4 (5)	574 ± 10.1 (5)	442 ± 33.4 (5)	c
End-Systolic Pressure (mmHg)	87.5 ± 6.06 (5)	92.1 ± 9.9 (5)	63.9 ± 6.31 (5)	c,e
End-Diastolic Pressure (mmHg)	9.46 ± 2.23 (5)	9.58 ± 2.26 (5)	10.3 ± 2.29 (5)	
Maximum dP/dt (mmHg/s)	7105 ± 960 (5)	10269 ± 1788 (5)	3473 ± 411 (5)	e
Minimum dP/dt (mmHg/s)	6646 ± 468 (5)	6713 ± 633 (5)	3290 ± 531 (5)	c,d,e
Tau (msec)	9.32 ± 1.17 (5)	8.11 ± 0.981 (5)	15.5 ± 3.04 (5)	
End-Diastolic Volume (μl)	37.2 ± 4.59 (5)	40 ± 3.89 (5)	60.1 ± 11 (5)	
End-Systolic Volume (μl)	24.6 ± 6.64 (5)	20.7 ± 5.5 (5)	53.6 ± 11.2 (5)	
Cardiac Output (μl/min)	11217 ± 1107 (5)	15716 ± 2654 (5)	8072 ± 1368 (5)	
PRSW (mmHg⋅μl/μl)	74.3 ± 7.46 (5)	78.6 ± 11.6 (5)	20.9 ± 4.25 (4)	

Significant testing by three-way ANOVA with genotype, oxygen therapy protocol, and hemodynamic protocol treatment as factors with Tukey post-hoc test. a; signifies difference between baseline and dobutamine with normoxic protocol in mdx mice; b: signifies difference between baseline and esmolol with normoxic treatment in mdx mice; c: signifies an overall genotype effect; d: represents an overall difference between normoxia and HBOT treatment; e: represents an overall difference between normoxia and hyperoxic treatment.

**Table 3:** HBOT C10 Hemodyanmic Data.

	Baseline	Dobutamine	Esmolol	Significance
Heart Rate (*bpm*)	444 ± 25.3 (6)	532 ± 25.7 (6)	406 ± 19.7 (5)	c
End-Systolic Pressure (mmHg)	92.5 ± 4.47 (6)	107 ± 4.14 (6)	81.3 ± 3.99 (5)	c
End-Diastolic Pressure (mmHg)	8.55 ± 0.864 (6)	9.11 ± 0.584 (6)	11.6 ± 0.75 (5)	
Maximum dP/dt (mmHg/s)	6411 ± 566 (6)	14436 ± 887 (6)	4395 ± 193 (5)	a
Minimum dP/dt (mmHg/s)	7242 ± 1059 (6)	12142 ± 1369 (6)	4628 ± 387 (5)	a,c,d
Tau (msec)	8.34 ± 1.02 (6)	5.92 ± 0.373 (6)	12 ± 0.415 (5)	
End-Diastolic Volume (μl)	44.7 ± 6.61 (6)	35.4 ± 6.49 (6)	56.1 ± 6.87 (5)	
End-Systolic Volume (μl)	30.3 ± 5.36 (6)	15.6 ± 3.64 (6)	42.2 ± 8.11 (5)	
Cardiac Output (μl/min)	8439 ± 975 (6)	12669 ± 2339 (6)	8808 ± 523 (5)	
PRSW (mmHg⋅μl/μl)	59.9 ± 6.79 (6)	76.8 ± 8.7 (6)	30.5 ± 11.4 (5)	

Significant testing by three-way ANOVA with genotype, oxygen therapy protocol, and hemodynamic protocol treatment as factors with Tukey post-hoc test. a; signifies difference between baseline and dobutamine with HBOT protocol in C10 mice; b: signifies difference between baseline and esmolol with HBOT treatment in C10 mice; c: signifies an overall genotype effect; d: represents an overall difference between normoxia and HBOT treatment; e: represents an overall difference between HBOT and hyperoxic treatment.

**Table 4:** HBOT mdx Hemodyanmic Data.

	Baseline	Dobutamine	Esmolol	Significance
Heart Rate (*bpm*)	503 ± 10.9 (8)	565 ± 10.5 (8)	397 ± 17.3 (7)	b,c
End-Systolic Pressure (mmHg)	93 ± 6.44 (8)	98.2 ± 4.91 (8)	63.6 ± 5.44 (7)	b,c
End-Diastolic Pressure (mmHg)	8.74 ± 1.02 (8)	8.17 ± 1.13 (8)	9.3 ± 0.966 (7)	
Maximum dP/dt (mmHg/s)	7974 ± 1293 (8)	11761 ± 1280 (8)	3355 ± 382 (7)	b
Minimum dP/dt (mmHg/s)	7817 ± 1288 (8)	8261 ± 1064 (8)	3676 ± 676 (7)	b,c,d
Tau (msec)	8.01 ± 0.529 (8)	6.95 ± 0.75 (8)	17.6 ± 5.87 (7)	
End-Diastolic Volume (μl)	45.3 ± 4.27 (8)	40.6 ± 5 (8)	49.7 ± 11 (7)	
End-Systolic Volume (μl)	29.1 ± 4.75 (8)	19.2 ± 3.59 (8)	40.9 ± 7.25 (7)	
Cardiac Output (μl/min)	11073 ± 652 (8)	14571 ± 1695 (8)	6367 ± 2095 (7)	
PRSW (mmHg⋅μl/μl)	66.4 ± 7.1 (8)	82 ± 9.27 (8)	30.8 ± 4.55 (7)	

Significant testing by three-way ANOVA with genotype, oxygen therapy protocol, and hemodynamic protocol treatment as factors with Tukey post-hoc test. a; signifies difference between baseline and dobutamine with HBOT protocol in mdx mice; b: signifies difference between baseline and esmolol with HBOT treatment in mdx mice; c: signifies an overall genotype effect; d: represents an overall difference between normoxia and HBOT treatment; e: represents an overall difference between HBOT and hyperoxic treatment.

**Table 5:** Hyperoxia C10 Hemodyanmic Data.

	Baseline	Dobutamine	Esmolol	Significance
Heart Rate (*bpm*)	452 ± 20.6 (7)	567 ± 12.2 (7)	403 ± 19.2 (6)	a,c
End-Systolic Pressure (mmHg)	93.6 ± 2.9 (7)	105 ± 4.54 (7)	82 ± 5.23 (6)	c,d
End-Diastolic Pressure (mmHg)	9.15 ± 1.74 (7)	7.84 ± 1.48 (7)	10.8 ± 2.13 (6)	
Maximum dP/dt (mmHg/s)	7540 ± 817 (7)	15042 ± 882 (7)	4740 ± 523 (6)	a,d
Minimum dP/dt (mmHg/s)	7714 ± 722 (7)	10937 ± 972 (7)	5049 ± 791 (6)	c,d
Tau (msec)	7.62 ± 0.856 (7)	5.97 ± 0.401 (7)	11.2 ± 1.41 (6)	
End-Diastolic Volume (μl)	43.7 ± 6.26 (7)	45.9 ± 5.67 (7)	54.3 ± 10 (6)	
End-Systolic Volume (μl)	28.7 ± 5.9 (7)	18.5 ± 1.34 (7)	43.7 ± 8.1 (6)	
Cardiac Output (μl/min)	9687 ± 271 (7)	18974 ± 3268 (7)	6259 ± 984 (6)	
PRSW (mmHg⋅μl/μl)	61.9 ± 7.64 (7)	85.4 ± 11.1 (7)	33.4 ± 4.11 (6)	

Significant testing by three-way ANOVA with genotype, oxygen therapy protocol, and hemodynamic protocol treatment as factors with Tukey post-hoc test. a; signifies difference between baseline and dobutamine with hyperoxic protocol in C10 mice; b: signifies difference between baseline and esmolol with hyperoxic treatment in C10 mice; c: signifies an overall genotype effect; d: represents an overall difference between hyperoxia and normoxic treatment; e: represents an overall difference between HBOT and hyperoxic treatment.

**Table 6:** Hyperoxia mdx Hemodynamic Data.

	Baseline	Dobutamine	Esmolol	Significance
Heart Rate (*bpm*)	490 ± 17.2 (8)	562 ± 6.9 (8)	399 ± 10.2 (8)	b,c
End-Systolic Pressure (mmHg)	93.1 ± 2.19 (8)	104 ± 2.59 (8)	69.7 ± 6.11 (8)	c,d
End-Diastolic Pressure (mmHg)	8.84 ± 1.22 (8)	8.61 ± 1.02 (8)	10.3 ± 0.771 (8)	
Maximum dP/dt (mmHg/s)	8478 ± 464 (8)	13460 ± 303 (8)	3974 ± 479 (8)	a,b,d
Minimum dP/dt (mmHg/s)	8402 ± 389 (8)	9418 ± 292 (8)	4464 ± 688 (8)	b,c,d
Tau (msec)	7.23 ± 0.61 (8)	6.5 ± 0.691 (8)	15.3 ± 3.9 (8)	
End-Diastolic Volume (μl)	38.6 ± 3.19 (8)	37.7 ± 4.93 (8)	50.1 ± 5.1 (8)	
End-Systolic Volume (μl)	25.4 ± 2.29 (8)	17.5 ± 1.52 (8)	41.2 ± 5.2 (8)	
Cardiac Output (μl/min)	9738 ± 749 (8)	14288 ± 3075 (8)	7382 ± 2017 (8)	
PRSW (mmHg⋅μl/μl)	66.1 ± 3.38 (8)	85.4 ± 3.63 (8)	40.3 ± 7.45 (8)	

Significant testing by three-way ANOVA with genotype, oxygen therapy protocol, and hemodynamic protocol treatment as factors with Tukey post-hoc test. a; signifies difference between baseline and dobutamine with hyperoxic protocol in mdx mice; b: signifies difference between baseline and esmolol with hyperoxic treatment in mdx mice; c: signifies an overall genotype effect; d: represents an overall difference between hyperoxia and normoxic treatment; e: represents an overall difference between HBOT and hyperoxic treatment.

**Figure 6:**
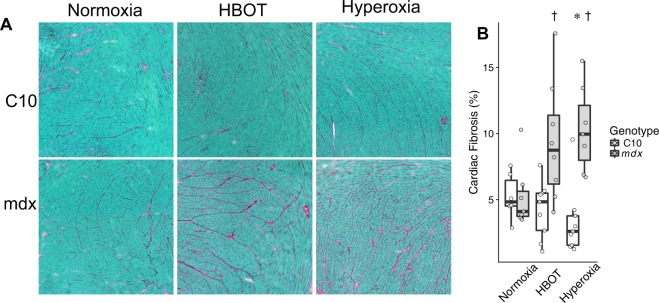
Cardiac fibrosis in C10 and mdx mice following 4 weeks of oxygen based treatment. Sirius red/fast green stain reveals increased collagen staining (red) in mdx hearts with both hyperbaric and hyperoxic conditions. Each image is 1 mm2. ANOVA with Tukey posttest was used to determine statistical significance. *Denotes a significant (P < 0.05) difference from mdx under normoxic conditions; ^†^Denotes a significant difference from corresponding C10 treatment group.

Dystrophic heart disease is associated with the progressive accumulation of fibrosis. The area of cardiac fibrosis was measured in hearts of mice following oxygen-based therapies. Under normoxic conditions, the young dystrophic mice used in this study had normal levels of fibrosis. Importantly, dystrophic mice exposed to either hyperbaric or hyperoxic displayed significantly greater areas of fibrosis than non-dystrophic mice exposed to the same conditions (Fig. 6). Analysis of hydroxyproline content of these hearts show an increase in mdx hearts subjected to hyperbaric therapy (27.9 ± 2.2 vs. 21.6 ± 1.7 µg hydroxyproline per mg of protein for mdx and C10 hearts respectively; p = 0.04). Interestingly, this assay does not demonstrate significant changes between the genotypes of the other treatment groups; 33.3 ± 3.7 in mdx hearts vs. 25.1 ± 3.2 in C10 hearts with normoxia and 16.9 ± 5.0 in mdx hearts vs. 22.3 ± 4.2 in C10 hearts exposed to hyperoxic conditions.

## Discussion

The primary motivating factor for these studies was to determine if hyperbaric therapy would have an effect on the pathophysiology of Duchenne muscular dystrophy (DMD) as manifested in the mdx mouse. Patients are already using this therapy in the absence of convincing data that it is effective, or even safe, for DMD patients. The results of this study demonstrate an increase in cardiac fibrosis area in dystrophic mice exposed to either of the experimental protocols and increased collagen content in mdx mice exposed to hyperbaric conditions. Furthermore, there is no evidence that the hyperbaric or hyperoxic exposure has any significant effect on skeletal muscle function with no significant changes observed throughout the 4 weeks of the protocol. These data are consistent with the lack of an effect of elevated oxygen exposure in chickens with muscular dystrophy^14^.

The protocol of 90 minutes of 1.3 atmosphere hyperbaric therapy, three times a day, seven days a week was designed to mimic a protocol that might be feasible with a small in-home hyperbaric chamber. This is the most likely form of hyperbaric therapy to be used by DMD patients as it has relatively low cost and does not require a special trip to a hyperbaric clinic. All mice in this study also received daily doses of ascorbic acid, similar to what is recommended to DMD patients, to limit any potential oxidative damage resulting from prolonged periods of hyperbaric or hyperoxic exposures. Given the highly controlled nature of this experiment, compliance to the protocol was 100%, which is likely greater than what would be achieved by DMD patients.

The current study has some notable limitations. First is the relatively modest disease present in the mdx mouse used in these studies. The mdx mouse was chosen for these studies as it provides a genetic model of DMD with other compensatory pathways intact and the mild phenotype was hypothesized to provide the best opportunity to observe any beneficial effects of hyperbaric or hyperoxic therapies. It is possible that the duration of the protocol was not sufficient to achieve the maximal effects of hyperbaric or hyperoxic therapy. However, if these therapies, as proposed, limited muscle damage or improved muscle recovery these effects should have been evident after four weeks of therapy, as any damaged muscle fibers would have been expected to recover by the end of the study.

The observed increase in cardiac fibrosis in dystrophic mice exposed to hyperbaric and hyperoxic conditions raises important questions regarding the safety of these approaches. One possible mechanism of this cardiac damage may result from stress the dystrophic mice feel in response to the raising of pressure or increases in oxygen. Perhaps the noise within the chambers causes an increase in sympathetic nervous system activity in the heart, increasing heart rate and cardiac contractility. The resulting increased work load may either damage the dystrophic heart or activate profibrotic signaling pathways resulting in the accumulation of fibrosis in the myocardium. Another potential mechanism is the effect of increased oxygen delivery on the respiratory drive of the dystrophic mice. Patients with chronic hypoventilation begin to rely on a degree of hypoxia to drive respiration, such that the sudden introduction of supplemental oxygen can decrease their respiratory efforts^15^. The result is that additional oxygen decreases ventilation resulting the development of respiratory acidosis, which may contribute to cardiac injury leading to the development of cardiac fibrosis. Based on the results of this study, DMD patients should be careful when using hyperbaric therapies to ensure that respiratory function is maintained during the course of the treatment period.

In summary, our findings suggest that 4 weeks of intermittent hyperbaric or hyperoxic exposure may result in an acceleration of cardiac disease and has no significant benefit on the skeletal muscle or respiratory disease process in mdx mice. The poor efficacy of these oxygen delivery based therapeutic approaches indicate that the levels of dissolved oxygen are not a significant contributor to the pathophysiology of the dystrophic mouse. The inability of these therapeutic approaches to provide any benefit to the mdx mouse raises important questions about the potential efficacy in DMD patients. The current lack of an effective therapy for many DMD patients results in patients and their families to seek alternative therapies, our data suggests that hyperbaric approaches, are unlikely to slow the course of disease progression and there is some potential that they may accelerate cardiac injury.

## Methods

### Animals

The mice used in these studies were single-housed males ranging from 4–6 months of age and taken from colonies of C57BL/10-SnJ (C10) and C57BL/10ScSn-DMD〈mdx〉/J mice maintained at the University of Minnesota. Genetic stability of the colony is maintained through the introduction of purchased breeders from Jackson Laboratories (Bar Harbor, ME) every 5 generations. While in the protocol of this study, all mice were given 12 mg/kg ascorbic acid in a small cookie (0.25 of a miniNilla wafer, Mondelez Global LLC), to mirror the antioxidant load in many patients using hyperbaric therapy. The mice readily consumed the cookie under the observation of study staff. All procedures were performed according to protocols reviewed and approved by the University of Minnesota Institutional Animal Care and Use Committee.

### Hyperbaric chamber

The hyperbaric chamber used was a Vitaeris 320 (OxyHealth LLC, Santa Fe Springs, CA), mice in standard mouse cages were placed within the chamber. The pressure within the chamber was controlled by custom designed computer-controlled valves and switches that allowed precise control of the timing of chamber pressure. Pressure within the chamber was determined by the pressure valve that is standard with the chamber and monitored using a barometric pressure sensor (BMP280 Pressure Sensor, Adafruit, New York, NY). Chamber carbon dioxide levels were monitored by using a K-30 CO_2_ sensor (SE-0018 CO2Meter.com). These signals were digitized using an Arduino R3 microcontroller (SainSmart, Lenexa, KS) connected via Bluetooth serial connection to a computer outside of the chamber. All electronic elements within the chamber were battery powered. During periods of normobaric pressures, the chamber air was exchanged using an air-pump (EcoPlus Commercial Air 7, Amazon, Seattle, WA) that maintained CO_2_ levels below 600 ppm.

### Hyperoxic chamber

Mice were placed in standard mouse cages; these cages were placed into a sealed cabinet. Oxygen levels were monitored using oxygen sensors (R17S, OxyCheq, Marianna, FL) digitized with Ardunino R3 microcontroller with 12-bit amplifier (ADS1015, Adafruit, New York, NY). Oxygen flow into the chamber was controlled by the same computer controlling the hyperbaric chambers, such that timing of experimental manipulations was synced. During periods of normoxia the chamber was flushed with room air to maintain a CO2 level below 600 ppm. Normoxic mice were housed in the same room as the HBOT and Hyperoxic treated mice, but were allowed to breathe room air.

### Whole-body plethysmography

Assessments of respiratory function were performed using the commercially available Buxco plethysmography system from Data Sciences International (Harvard Bioscience, Inc., Holliston, MA). Briefly, mice were placed in the plethysmography chamber following the first hyperbaric/hyperoxic period of the day. Mice were left in the chamber for 90 minutes during which time they were monitored by closed-circuit camera. Quality plethysmography data is obtained once the mice have fallen asleep, thus maintaining an undisturbed state was particularly important. Following completion of the measurement period, mice were returned to their home cages and returned to their treatment chambers.

### Treadmill protocol

On days for which treadmill protocols were scheduled, mice were removed from their treatment chambers after the first hyperbaric/hyperoxic period and placed in one of the lanes of a Columbus Instruments rodent treadmill (Columbus, OH). Prior to initiation of the studies mice were trained by being placed on the flat treadmill running a 5 m/min for 10–15 minutes. During the study, the treadmill was oriented such that mice ran down a 15° slope. Initial speed was set to 5 m/min for 5 minutes, then increased 1 m/min every minute until a maximum speed of 20 m/min was obtained. Mice were continued until exhaustion such that mice could no longer be encouraged to get on the treadmill and did not attempt to escape when removed from the treadmill lane.

### Grip strength

Grip strength was assessed using the grip strength meter (Columbus Instruments, Columbus, OH). Overall muscle strength was assessed by taking the maximum tension produced from ten repeated pulls on the grip bar. This tension was then corrected for the body weight of the mouse.

### Muscle morphology measurements

Quadricep cryosections were stained with WGA, goat-anti-mouse IgG, and DAPI to assess fiber morphology, acute muscle fiber injury, and nuclear localization. Image montages at 10x magnification were analyzed using a custom script in FIJI. Briefly, this script used the WGA channel to identify the boarders of fibers. A filter was applied to remove fibers that were not oriented in a cross-sectional plane. Central nucleation was determined by examining the DAPI signal within the central core of each fiber. For each mouse between 960 and 6049 fibers were assessed. Fiber cross sectional area distribution was calculated and plotted using R with ggplot^16–18^.

### Hemodynamic measurements

Cardiac function was assessed by cardiac catheterization using pressure-volume conductance catheter (Transonic Systems, Ithaca, NY) and previously described^12,13,19–21^. Briefly, mice were anesthetized using isoflurane, ventilated, and the heart was exposed via thoracotomy. The catheter was inserted through an apical stab incision and positioned within the ventricle. A jugular catheter was placed, and the mouse allowed to stabilize following infusion of 10% albumin. Cardiac contractile reserve was assessed through the intravenous infusion of 20 µg/kg/min dobutamine and basal adrenergic tone was determined by the infusion of 250 µg/kg/min esmolol.

### Fibrosis determination

*Histological:* At the time of euthanasia, hearts and muscles were collected and immediately frozen in liquid nitrogen cooled isopentane. These hearts were cryo-sectioned and stained with Sirius red/fast green as described previously^10,22^. Fibrosis was quantified in a blinded manner using ImageJ^23^. Briefly, 4x montages were made from short-axis cross sections of sirius red/fast green stained heart, diaphragm, and quadricep sections. The red staining collagen areas and total muscle area were manually determined using the color thresholding tool in ImageJ. The percentage of fibrosis was determined by calculating the number of positive red pixels divided by the total number of muscle pixels. *Biochemical:* Portion of hearts were homogenized in buffer (Tris-HCl: 20 mM, NaCl: 150 mM, Na-Pyrophosphate: 2.5 mM, EDTA: 1 mM, SDS: 1%, Leupeptin: 0.5 µg/ml, Pepstatin: 0.6 µg/ml, and PMSF: 0.1 mM). Protein concentration determined using the BCA assay from Thermo-Fisher. Hydroxyproline content was determined using previously described methods^24^.

### Statistics and analysis

All collected data was analyzed using R^25^. All comparisons were analyzed by ANOVA with a Tukey post-hoc test to control for multiple comparisons.
